# An Integrated Local Classification Model of Predicting Drug-Drug Interactions via Dempster-Shafer Theory of Evidence

**DOI:** 10.1038/s41598-018-30189-z

**Published:** 2018-08-07

**Authors:** Jian-Yu Shi, Xue-Qun Shang, Ke Gao, Shao-Wu Zhang, Siu-Ming Yiu

**Affiliations:** 1 School of Life Sciences, Northwestern Polytechnical University, Xi’an, 710072 China; 2 School of Computer Science, Northwestern Polytechnical University, Xi’an, 710072 China; 3 School of Automation, Northwestern Polytechnical University, Xi’an, 710072 China; 4 Department of Computer Science, The University of Hong Kong, Hong Kong, 999077 China

## Abstract

Drug-drug interactions (DDIs) may trigger adverse drug reactions, which endanger the patients. DDI identification before making clinical medications is critical but bears a high cost in clinics. Computational approaches, including global model-based and local model based, are able to screen DDI candidates among a large number of drug pairs by utilizing preliminary characteristics of drugs (e.g. drug chemical structure). However, global model-based approaches are usually slow and don’t consider the topological structure of DDI network, while local model-based approaches have the degree-induced bias that a new drug tends to link to the drug having many DDI. All of them lack an effective ensemble method to combine results from multiple predictors. To address the first two issues, we propose a local classification-based model (LCM), which considers the topology of DDI network and has the relaxation of the degree-induced bias. Furthermore, we design a novel supervised fusion rule based on the Dempster-Shafer theory of evidence (LCM-DS), which aggregates the results from multiple LCMs. To make the final prediction, LCM-DS integrates three aspects from multiple classifiers, including the posterior probabilities output by individual classifiers, the proximity between their instance decision profiles and their reference profiles, as well as the quality of their reference profiles. Last, the substantial comparison with three state-of-the-art approaches demonstrates the effectiveness of our LCM, and the comparison with both individual LCM implementations and classical fusion algorithms exhibits the superiority of our LCM-DS.

## Introduction

Drug-drug interactions (DDIs) may occur unexpectedly when drugs are co-prescribed. The identification of DDIs is difficult in the process of drug development^1^ because of both a small number of participants and a large number of drug pairs in clinical screening. Thus, a minority of DDIs could be usually identified in the clinical trial phase while a majority of them are only reported after the co-prescription of multi-drugs are made. On the one hand, DDIs would trigger adverse drug reactions, such as efficacy reduction and toxicity increment, such that the patients, who are treated with multiple drugs, are led into unsafe and even incorrect medications^2–5^. The broadcasting of adverse effects caused by DDIs cannot be neglected, because the number of potential DDIs is rising with the power of two along with the increasing number of approved drugs. On the other hand, the identification of DDIs is one of the crucial steps towards finding synergistic drug combinations^6^, which further cover the issues about drug targets, drug resistance and drug sensitivity^7^.

Consequently, it is imperative to identify DDIs before multi-drug co-prescriptions are made. However, experimental approaches (e.g. testing cytochrome P450^8^ or transporter-associated interactions^9^) are performed under the consideration of animal welfare with high costs in both money and time. In contrast, computational approaches can help screen DDI candidates among a large number of drug pairs with much lower costs. They have winning interests from both academy and industry recently^10–12^.

Text-mining based computational approaches apply the techniques of text-mining to detect DDIs recorded in diverse text sources, such as scientific literatures^13–15^, electronic medical records^16^, and the Adverse Event Reporting System of FDA. These approaches are particularly helpful when building a DDI database. However, they cannot predict or alert newly potential DDIs for multi-drug treatment, because they depend on the evidence, which reports the DDIs found in clinical treatments.

Many works in other areas have demonstrated that the utilization of pre-existing knowledge is promising to build a predictive model with the advantages of both low cost and good effectiveness. For example, the genes identified *in silico* as cancer prognostic biomarkers^17^ show their strong correlations with cancers, such that they have been widely used to build predictive models for diverse cancer-related alerts (e.g. survival of patients under PIK3CA-mutated Breast cancer^18^, tumor clinical phenotypes^19^, and recurrence of colorectal cancer^20^). For another example similar to DDI prediction, the observation that similar drugs tend to interact with similar targets inspires the development of diverse models for predicting drug-target interaction^21–24^. Analogously, by leveraging pre-existing drug properties (e.g. chemical structures^25^, targets^26^, drug classification codes^27^ and side effects^28^) which can be acquired before multiple drugs are co-prescribed, similarity-based computational approaches are able to provide predictive models for deducing potential DDIs. These approaches can be grouped as global model-based (e.g. global classification-based^27^) and local model-based(e.g. naïve similarity-based approach^25^ and network recommendation-based^28^). They enable drug safety professionals to screen potential DDIs fast so as to help make appropriate clinical multi-drugs treatments.

Similarity-based approaches usually hold diverse assumptions. Global classification model-based approaches (GCM) accept that DDIs are globally distinct to non-DDIs^27^. After treating DDIs and non-DDIs as positive and negative instances respectively, it trains a classifier to perform DDI prediction for new drugs. However, GCM neglects the topological relationship between DDIs and non-DDIs. Local model-based approaches (e.g. Naïve similarity-based approach^25^ and label propagation^28^) consider such a relationship to some extent and utilize the local topology of a DDI network. They usually run faster than GCM. Nevertheless, both of them have the intrinsic degree-induced bias that greatly tends to generate a high confidence of being a DDI to the pair of a newly-given drug having no known DDI and a known drug having many DDIs. Even though such a bias is probably useful to DDI prediction, it deserves a better utilization or relaxation.

Furthermore, though current approaches always utilize a single model to perform DDI prediction, they lack the effective combination of multiple predictions generated by different predictors/models (e.g. classifiers). The fact that different models may fit different data characteristics of pre-existing drug properties/knowledge should be considered.

To address the abovementioned issues, we first propose a novel local classification-based model (LCM) under the assumption that similar drugs tend to interact with the same drugs. Then, we design a novel supervised fusion algorithm based on the Dempster-Shafer theory of evidence (LCM-DS), which aggregates the results from different LCMs. Last, in the scenario of predicting potential DDIs for new drugs, the results demonstrate that the proposed LCM is superior to three state-of-the-art approaches greatly and its ensemble version LCM-DS outperforms both three individual LCM implementations and five classical fusion algorithms.

## Methods

### Local Classification Model

Given m drugs, *D* = {*d*_*i*_}, *i* = 1, 2, ..., *m*, of which each has at least one DDI with others. Their pairwise interactions are accordingly arranged into an *m* × *m* binary symmetric matrix **A**_*m*×*m*_ = {*a*_*ij*_}, in which *a*_*ij*_ = *a*_*ji*_ ∈ {0, 1}, *a*_*ij*_ = 1 if the interaction between *d*_*i*_ and *d*_*i*_ occurs, *a*_*ij*_ = 0 otherwise. Moreover, their pairwise similarities are organized into another *m* × *m* positive symmetric matrix **S**_*m*×*m*_ = {*s*_*ij*_}, where ${s}_{ij}\in {{\mathbb{R}}}_{+}$ denotes the similarity between *d*_*i*_ and *d*_*j*_. For a newly give drug *d*_*x*_, which has no interaction with any drugs in *D*, its pairwise similarities to all *d*_*i*_ are also organized into a vector ${{\bf{S}}}_{1\times m}^{x}$.

Our problem is to infer how likely the new drug *d*_*x*_ interacts with the drugs in *D* and it is represented as a set of local drug-specific classifications as follows.

**Figure 1:**
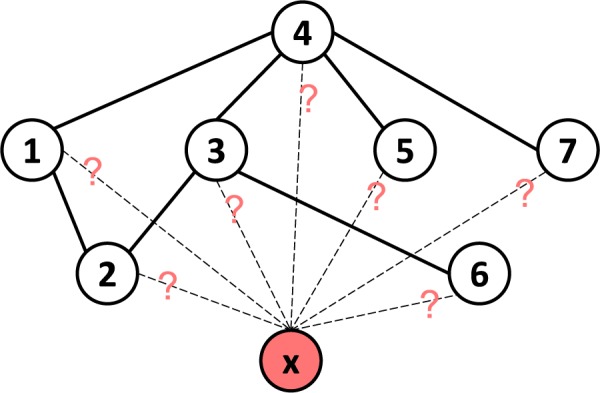
Illustration of LCM predicting DDI for a newly given drug. Nodes are drugs. The hollow nodes are known drugs and the solid lines between them denote their interactions. The node filled with red is the newly given drug. Our problem is to determine which drugs it is likely to interact with.

In the local classification specific to drug *d*_*i*_ in *D*, we first label the drugs interacting with *d*_*i*_ as positive instances and other drugs in *D* as negative instances. For example in Fig. 1, when predicting how likely *d*_*x*_ interacts with *d*_4_, we assign *d*_1_, *d*_3_, *d*_5_ and *d*_7_ with positive labels, *d*_2_ and *d*_6_ with negative labels respectively. Then, we train a classifier *C* specific to *d*_*i*_ by the labels **L** of the drugs and their pairwise similarity matrix **S**_*m*×*m*_. Finally, we apply the well-trained classifier *C* on the unlabeled instance *d*_*x*_ to obtain its label. Generally, the classifier simply outputs a single label to denote a positive or a negative instance. Because we need to know how likely *d*_*x*_ interact with a specific drug in *D*, the classifier is required to output a 2-dimensional decision profile vector ${{\bf{y}}}^{x}=C(x)=[{p}_{+},{p}_{-}]$, where *p*_+_, *p*_−_ ∈ [0, 1] are the probabilities of *d*_*x*_ being a positive instance and a negative instance respectively, and they satisfy *p*_+_ + *p*_−_ = 1.

The proposed LCM has a faster training and a requirement of less memory than GCM^27^ because the number of instances handled by LCM is the number of drugs but not the number of drug pairs, which is usually huge, handled by GCM. Compared with NS^25^ and LP^28^, LCM is able to minimize their intrinsic degree-induce bias because the prediction for a new drug depends only on the distributions of positive instances and negative instances in feature or similarity space (see also Section 3.2).

### Similarity Calculation

Drugs are popularly represented as binary profiles according to diverse drug properties, such as fingerprints of chemical structures and keyword occurrence lists of side effects. In the binary profile of a drug, each entry denotes the presence or absence of one of its concerned properties by 1 or 0 respectively. A classical similarity measure widely adopted by former works is Jaccard Index (also called as Tanimoto coefficient). Technically, the pairwise similarity between two drugs is defined as *S*_*Jaccard*_(*i*, *j*) = |**f**_*i*_ ∩ **f**_*j*_|/|**f**_*i*_ ∪ **f**_*j*_|, where the numerator is the number of common presence entries between **f**_*i*_ and **f**_*j*_ while the denominator is the number of presence entries in their binary union. Once a similarity matrix is given, it can be exploited to train a classifier and make the prediction.

### Classifiers

Except for similarity, the classifier is another crucial factor in classification. When implementing LCM, we considered three classifiers, multi-label K nearest neighbors (MLKNN)^23^, Regularized Least Squared classifier (RLS)^24,29^ and Support Vector Machines (SVM)^30^, of which all can accept the form of similarity matrix as their input. Their brief introductions are shown in the following respectively. In addition, we refer to drugs as instances in the context of classification.

- MLKNN: Denote *N*^*j*^(*x*, *K*) as the set of K nearest neighbors of instance *d*_*x*_, *n*^*j*^(*x*, *K*) as the number of neighbors interacting with *d*_*j*_ (having positive labels) among *N*^*j*^(*x*, *K*), and ${p}_{x}^{j}$ as the probability that *d*_*x*_ interacts with *d*_*j*_ (a positive label). When *d*_*x*_ is a testing instance, ${p}_{x}^{j}\in [0,1]$ defines its confidence score of being a positive instance as follows
  1 $${p}_{x}^{j}=\frac{\Pr [{y}^{j}=1]\cdot \Pr [{n}^{j}(x,K)=k|{y}^{j}=1]}{{\sum }_{t=\{0,1\}}\Pr [{y}^{j}=t]\cdot \Pr [{n}^{j}(x,K)=k|{y}^{j}=t]}$$
  where $\Pr [{y}^{j}=t]$ is the prior probability of an instance having label *t* and $\Pr [{n}^{j}(x,K)=k|{y}^{j}=t]$ is the probability of *d*_*x*_ having *k* neighbors under the condition of a positive/negative label with respect to *d*_*j*_. Two prior probabilities can be directly estimated by $\Pr [{y}^{j}=1]\approx (1+{c}^{j})/(m+2)$ where *c*^*j*^ is the number of drugs interacting with *d*_*j*_ and $\Pr [{y}^{j}=0]=1-\Pr [{y}^{j}=1]$. The conditional probability can be estimated by
  2 $$\Pr [{n}^{j}(x,K)=k|{y}^{j}=t]=\frac{1+{\sum }_{i}\,B[{y}^{j}(i)=t\,\& \,{n}^{j}(x,K)=k]}{(K+1)+{\sum }_{k^{\prime} =0}^{K}{\sum }_{i}\,B[{y}^{j}(i)=t\,\& \,{n}^{j}(x,K)=k^{\prime} ]}$$
  where *y*^*j*^(*i*) = *t* is the *i*-th drugs having label *t*, *B*[*S*] = 1 if statement *S* is correct and *B*[*S*] = 0 otherwise. Totally, we generate two probability tables, which account for positive instances and negative instances respectively. Each of them contains *K* + 1 probability entries, which correspond to the *K* + 1 possible values with respect to *n*^*j*^(*x*, *K*) = 0, 1, ..., *K* respectively.
   Note that, for a queried instance, the theoretical version of MLKNN uses the distances to other instances to find its top K neighbors^31^, while our input is a set of pairwise similarities between instances (organized into a similarity matrix). To bridge the gap, we need to turn similarities into distances by two points. First, the smaller the distance between two instances is, the greater their similarity is. In addition, the value of distance should be non-negative. Thus, the distance between two instances is finally defined as by 1- their similarity, such that the K nearest neighbors of an instance are just the top K most similar instances to it^23^.
- RLS: Let **D** be the set of the training instances (drugs), *d*_*x*_ be the testing instance, **Y**_*j*_ = **A**(:, *j*) be the *m* × 1 class label vector of training instances which are specific to drug *d*_*j*_ and correspond to the j-th column of the interaction matrix, and **K**(**X**_1_, **X**_2_) be the kernel matrix, which reflects the pairwise similarities between two groups of drugs. Specifically, **K**(**D**, **D**) = **S**_*m*×*m*_, which contains the pairwise similarities of **D**, and ${\bf{K}}({d}_{x},{\bf{D}})={{\bf{S}}}_{1\times m}^{x}$, which contains the pairwise similarities between *d*_*x*_ and *m* training drugs. RLS classifier is an elegant linear system, which has the order equal to the number of training instances^24^. The trained RLS classifier outputs the confidence score *f*_*j*_(*d*_*x*_) of how likely a given new drug *d*_*x*_ interacts with drug *d*_*j*_ as follows,
  3 $${f}_{j}({d}_{x})={\bf{K}}({d}_{x},{\bf{D}}){({\bf{K}}({\bf{D}},{\bf{D}})+\alpha {\bf{I}})}^{-1}{{\bf{Y}}}_{j}$$
  where ***I*** is the *m* × *m* identity matrix and *α* is the regularization parameter (usually equal to 0.5) to prevent overfitting.
- SVM: Similar to RLS, SVM is also a kernel-based classifier, which can perform the highly non-linear classification as a linear classification by kernel trick^30^. Usually, the training of a binary *d*_*j*_-specific SVM depends on the solution of the following optimization problem

4 $$\begin{array}{c}\mathop{{\rm{\max }}}\limits_{\alpha }\sum _{k=1}^{M}{\alpha }_{k}^{j}-\frac{1}{2}\sum _{k=1}^{M}\sum _{i=1}^{M}{\alpha }_{k}^{j}{\alpha }_{i}^{j}{y}_{k}^{j}{y}_{i}^{j}{\bf{K}}({d}_{k},{d}_{i}),\\ s.\,t.\,0\le {\alpha }_{k}^{j}\le \gamma \,{\rm{and}}\,{\sum }_{k=1}^{M}{\alpha }_{k}^{j}{y}_{k}^{j}=0\end{array}$$

where ${y}_{k}^{j}\in \{0,1\}$ is the *d*_*j*_-specific label of the training drug *d*_*k*_, *M* is the number of the training instances, ***K*** is the kernel function, *γ* is the tunable parameter to reflect the trade-off between the training error and the margin of separation, and the variable ${\alpha }_{k}^{j}$ to be solved is the *d*_*j*_-specific weight of *d*_*k*_. Once the training of SVM is done, for the given testing instance *d*_*x*_, it outputs the confidence score of how likely a given new drug *d*_*x*_ interacts with drug *d*_*j*_ by a linear operation as follows,

5 $${s}_{j}({d}_{x})=\sum _{k=1}^{M}{\alpha }_{k}^{j}{y}_{k}^{j}K({d}_{k},{d}_{x})+b.$$

The abovementioned three classifiers shall be taken as the member classifiers when performing the integration of classifiers for DDI prediction in the next section.

### Classifier fusion

In the context of classifier fusion, our problem is restated as the inference of how likely a given drug *d*_*x*_ interacts with a specific drug *d*_*j*_ by combining the evidences generated by a group of classifiers.

Formally, given *m* training instances *X* = {*x*_*i*_}, *i* = 1, 2, ..., *M* with their labels *l*_*i*_ ∈ {1, 2, ..., *K*} where *K* is the number of classes (e.g. *K* = 2 in our problem). N classifiers {*C*_*n*_} *n* = 1, ..., *N* are trained by their similarity matrices and their labels. Classifier *C*_*n*_ generates a *K*-dimensional decision profile ${{\bf{y}}}_{n}^{x}\in {\Re }^{K}$ for an unlabeled instance *x*.

**Figure 2:**
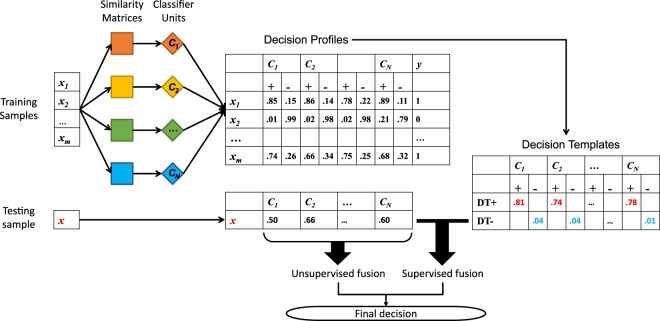
Architecture of classifier fusion.

Denote ${e}_{k}({{\bf{y}}}_{n}^{x})$ as the evidence supporting the proposition “classifier *C*_*n*_ thinks that *x* is of class *k*”. Classifier fusion combines the evidences $\{{e}_{k}({{\bf{y}}}_{n}^{x})\}$ to make a final decision of how likely *x* is of class *k*.The general architecture of classifier fusion is shown in Fig. 2.

The fusion rules of evidences can be categorized into unsupervised and supervised rules. Unsupervised rules combine those output evidences of *x* directly by arithmetical operations, such as Average, Product, Maximum and Minimum. For a given unlabeled instance *x*, the above four unsupervised rules are defined as follows

6 $$\begin{array}{c}{e}_{k}^{ave}({{\bf{y}}}^{x})=\sum _{n=1}^{N}{e}_{k}({{\bf{y}}}_{n}^{x})/N,{e}_{k}^{prod}({{\bf{y}}}^{x})=\prod _{n=1}^{N}{e}_{k}({{\bf{y}}}_{n}^{x}),\\ {e}_{k}^{\max }({{\bf{y}}}^{x})=\mathop{{\rm{\max }}}\limits_{n=1,\mathrm{...},N}\{{e}_{k}({{\bf{y}}}_{n}^{x})\},{e}_{k}^{\min }({{\bf{y}}}^{x})=\mathop{\min }\limits_{n=1,\mathrm{...},N}\{{e}_{k}({{\bf{y}}}_{n}^{x})\}.\end{array}$$

While supervised rules need to generate a training profile by the evidences of the training instances, and integrate the training profile with the evidences of *x* generated by classifiers to make the combined evidence of *x*. Decision Template is a popular supervised rule^32^ and has been applied in other related areas (e.g. drug-target interaction prediction^33^). It combines the evidence of *x* from different classifiers by

7 $${e}_{k}({{\bf{y}}}^{x})=1-\frac{1}{N}\sum _{n=1}^{N}{({{\bf{y}}}_{n}^{x}-{\bf{D}}{{\bf{T}}}_{k})}^{2}$$

where ${\bf{D}}{{\bf{T}}}_{k}=\frac{1}{{N}_{k}}\sum _{i=1}^{{N}_{k}}{{\bf{y}}}_{n}^{{x}_{i}}$ is the decision template of class k, which is generated from the training instances. In details, *x*_*i*_ denotes the instances having class label *l*_*i*_ = *k* in *X*, ${{\bf{y}}}_{n}^{{x}_{i}}$ is their discriminating profile, and *N*_*k*_ is the number of such instances and ${\sum }_{k=1}^{K}{N}_{k}=M$.

In the next section, we should introduce a novel supervised fusion based on the Dempster-Shafer Theory of Evidence.

### Dempster-Shafer Theory of Evidence

When representing and combining measures from different sources (e.g. the decisions of multiple classifiers), the Dempster–Shafer (DS) theory of evidence provides a better frame of discernment than the Bayesian theory by generalizing Bayesian reasoning^34^. This theory defines a set of mutually exhaustive and exclusive atomic hypotheses Θ = {*θ*_1_, ..., *θ*_*K*_}, and its power set 2^Θ^ that contains the empty set ∅, Θ itself and other subsets of Θ. For the *K*-dimensional decision profile generated by classifier *C*_*n*_ generates, each hypothesis *θ*_*k*_ represents that “${{\bf{y}}}_{n}^{x}$ is of class *k*.” In the case of binary classification, Θ = {+, −} and its power set 2^Θ^ = {∅, {+}, {−}, Θ}.

The DS theory of evidence also assigns a belief mass function, called Basic Belief Assignment (BBA), to each element in 2^Θ^. The BBA function ${\rm{m}}:{{\rm{2}}}^{{\rm{\Theta }}}\to [0,1]$ is defined as

8 $${\rm{m}}(\varnothing )=0\,\,{\rm{and}}\,\sum _{A\in {2}^{\Theta }}{\rm{m}}(A)=1$$

where *A* is named as a composite hypothesis, which may contain an individual atomic hypothesis or multiple atomic hypotheses, and it satisfies ${\rm{m}}(A)+{\rm{m}}(\overline{A})\le 1$. In classification, *A* represents that “${{\bf{y}}}_{n}^{x}$ is only of composite class *A* but none of its subsets” such that the conflict between evidences can be modeled. The BBA function ${\rm{m}}(A)$ reflects how many relevant and available evidences support the composite hypothesis. The theory provides a combination rule ${\rm{m}}={{\rm{m}}}_{1}\,\oplus \,{{\rm{m}}}_{2}$ for two BBAs m_1_ and m_2_. It is defined as:

9 $${\rm{m}}(A)={Z}^{-1}\sum _{B\cap C=A}{{\rm{m}}}_{1}(B)\cdot {{\rm{m}}}_{2}(C)$$

where $$Z=\sum _{B\cap C\ne \varnothing }{{\rm{m}}}_{1}(B)\cdot {{\rm{m}}}_{2}(C)$$.

Furthermore, this theory defines a belief function ${\rm{Bel}}:{{\rm{2}}}^{{\rm{\Theta }}}\to [0,1]$, which is the sum of all the masses of subsets *B* of the set of interest *A* and satisfies $\mathrm{Bel}(A)=\sum _{B\subseteq A}{\rm{m}}(B)$. Suppose that a simple support function Bel satisfies $\mathrm{Bel}({\rm{\Theta }})=1$ and its focus *F* ⊆ Θ. We have $\mathrm{Bel}(A)=0\,{\rm{if}}\,F\not\subset A$ and $\text{Bel}(A)=b$ if *F* ⊆ *A* & *A* ≠ Θ, where *b* is called Bel’s degree of support.

Therefore, a BBA can be considered as a generalization of a probability density function, while a Bel is a generalization of a probability function. Obviously, if *A* is an atomic hypothesis, ${\rm{Bel}}(A)={\rm{m}}(A)$. Besides, in the case of ${\rm{m}}({\theta }_{i})\ne 0$ for all the atomic hypotheses and ${\rm{m}}(A)=0$ for all the composite hypotheses, DS theory would simplify itself as the Bayesian probability theory.

### DS-Based Fusion

Our problem is now to predict how likely a given drug *d*_*x*_ interacts with a specific drug *d*_*j*_ according to the evidences generated by N classifiers. Inspired by Rogova’s work^35^, we consider the entry accounting for posterior probability of each class in the decision profile vector as a BBA (Equation 8) and design a novel DS-based fusion algorithm to address this problem in the following.

Define the reference profile ${{\bf{R}}}_{k}^{n}$ w.r.t class *k* and classifier *C*_*n*_ as the mean vector of a set of decision profile vectors $\{{C}_{n}({x}_{k}^{trn})\}$, where $\{{x}_{k}^{trn}\}$ are the training instances belonging to class *k*. Class-conditional probability distributions for all *K* classes can be estimated by both intra-class and inter-class distances between the decision profile vectors of instances and the class-specific reference profiles^36^. Thus, the reference profiles can largely reflect the abilities of *C*_*n*_ in classification.

Define a function ${s}_{k}^{n}=\varphi ({{\bf{R}}}_{k}^{n},{{\bf{y}}}_{n}^{x})$ as the likelihood measure of the decision profile ${{\bf{y}}}_{n}^{x}$ w.r.t classifier *C*_*n*_ and class *k*. It measures the evidence that supports the hypothesis *θ*_*k*_, while other measures $\{{s}_{j}^{n}\}$ where *j* ≠ *k* jointly represent the evidence which opposes *θ*_*k*_ or supports its negation $\overline{{\theta }_{k}}$.

Because the combined evidence ${e}_{k}({{\bf{y}}}^{x})$ finally indicates how likely the given instance *x* is of class *k*, we let ${s}_{k}^{n}$ reflect two aspects of instance *x*, ${y}_{n}^{x}(k)$ and $||{{\bf{R}}}_{k}^{n}-{{\bf{y}}}_{n}^{x}||$, w.r.t class *k*. The former is the posterior probability output by classifier *C*_*n*_ while the latter defines the proximity between the posterior probability profile and the reference output profile of class *k* for classifier *C*_*n*_. The norm in $||{{\bf{R}}}_{k}^{n}-{{\bf{y}}}_{n}^{x}||$ can be any form, such as L2-norm. Therefore, ${s}_{k}^{n}$ can be defined as follows,

10 $${s}_{k}^{n}=\frac{{y}_{n}^{x}(k)\cdot \exp (\,-\,||{{\bf{R}}}_{k}^{n}-{{\bf{y}}}_{n}^{x}||)}{\sum _{i=1}^{K}\,{y}_{n}^{x}(i)\exp (\,-\,||{{\bf{R}}}_{i}^{n}-{{\bf{y}}}_{n}^{x}||)}$$

The likelihood ${s}_{k}^{n}$ can be treated as a simple support function with focus *θ*_*k*_ and its value is just the supporting degree for the focus. Therefore, the BBAs of classifier *C*_*n*_ for focus *θ*_*k*_ specific to class *k* can be defined as

11 $${{\rm{m}}}_{k}^{n}({\theta }_{k})={s}_{k}^{n},{{\rm{m}}}_{k}^{n}(\overline{{\theta }_{k}})=0,{{\rm{m}}}_{k}^{n}({\rm{\Theta }})=1-{s}_{k}^{n}$$

On the opposite side, there are multiple measures $\{{s}_{j}^{n}\}$ where *j* ≠ *k* jointly measure how well the evidence $\overline{{\theta }_{k}}$ is supported. Thus, a combination of the simple support functions accounting for $\{{s}_{j}^{n}\}$ with focus $\overline{{\theta }_{k}}$ is need to define the BBAs. Obviously, the combined degree of support is $1-{\prod }_{j\ne k}(1-{s}_{j}^{n})$. Thus, the corresponding BBAs of classifier *C*_*n*_ for focus $\overline{{\theta }_{k}}$ specific to class *j* ≠ *k* are

12 $${{\rm{m}}}_{\overline{k}}^{n}({\theta }_{k})=0,{{\rm{m}}}_{\overline{k}}^{n}(\overline{{\theta }_{k}})=1-{\prod }_{i\ne k}(1-{s}_{i}^{n})\,,{{\rm{m}}}_{\overline{k}}^{n}({\rm{\Theta }})={\prod }_{j\ne k}(1-{s}_{j}^{n}).$$

Then, according to the combination rule of BBA (Equation 9), the evidence ${e}_{k}({{\bf{y}}}_{n}^{x})={{\rm{m}}}^{n}={{\rm{m}}}_{k}^{n}\oplus {{\rm{m}}}_{\overline{k}}^{n}$ supporting *θ*_*k*_ with respect to classifier *C*_*n*_ and class *k* is defined as:

13 $${e}_{k}({{\bf{y}}}_{n}^{x})=\frac{{s}_{k}^{n}\cdot {\prod }_{j\ne k}(1-{s}_{j}^{n})}{1-{s}_{k}^{n}\cdot [1-{\prod }_{j\ne k}(1-{s}_{j}^{n})]}$$

Last, the combination of the evidences generated by N classifiers w.r.t class k is defined as

14 $${e}_{k}({{\bf{y}}}^{x})=Z{\prod }_{1}^{N}{w}_{k}(n){e}_{k}({{\bf{y}}}_{n}^{x}),$$

where Z is the normalizing constant, and *w*_*k*_(*n*) is the weight of classifier *C*_*n*_ for class *k* among all the classifiers and is defined as

15 $${w}_{k}(n)=|{r}_{k}^{n}(k)-\sum _{j\ne k}{r}_{j}^{n}(k)|\cdot |{r}_{k}^{n}(k)-\sum _{j\ne k}{r}_{k}^{n}(j)|.$$

In details, *w*_*k*_(*n*) consists of a reference-between class specificity and a reference-within class specificity w.r.t class *k* and classifier *C*_*n*_, where ${r}_{p}^{n}(q)$ is the *q*-th element of the reference profile w.r.t class *p* generated by classifier *C*_*n*_. The first specificity term indicates how dominant the reference value of class *k* in the reference profile of class *k* is to those in the reference profiles of other classes, while the second one reflects how dominant the reference value of class *k* is to other values in the reference profile of class *k*.

In summary, when combining the outputs of multiple classifiers for an unlabeled instance under DS theory, our approach, LCM-DS, considers three aspects, including its direct outputs of classifiers (posterior probabilities), the difference (or proximity) between its outputs and the reference outputs of the training instances, and the class-specific weights w.r.t classifiers.

### Declaration

A preliminary version of this work has been published as an extended abstract (DOI: 10.1109/BIBM.2016.7822571).

### Data availability

Both the dataset and the codes of our LCM-DS can be freely downloaded from https://github.com/JustinShi2016/ScientificReports2018.

## Experiments and Results

### Settings

To validate the effectiveness of our approach, we adopted the DDI dataset in Zhang *et al*.’s work^28^, which contains 569 drugs and 52,416 pairwise interactions between them. The original work also provides three similarity matrices, derived from PubChem fingerprints of drug chemical structures^37^, a set of keywords of side effect recorded in SIDER^38^, as well as a list of medical terms of off-label side effects^39^ respectively. More details are shown in the original work^28^. We directly adopted their average as the final similarity matrix, which is used to train predictive models.

Though there are several implementations of SVM, we selected LibSVM^30^ because of its fast running as well as convenient usage. By regarding the similarity matrix as the pre-computed kernel matrix, we have only one tunable parameter, the cost *C*, of LibSVM. We investigated how *C* influences the prediction by simply tuning its value from a recommend list {0.125, 0.25, 0.5, 1, 2, 4, 8, 16, 32, 64, and 128} one by one. The predictions of DDI under 50% hold-out cross validation(CV) with 50 repetitions showed that the value of *C* doesn’t influence the prediction substantially, i.e., the method is quite robust in terms of these variations. For simplicity, we set *C* = 1 when training a LibSVM model in all the subsequent experiments. Likewise, we set the regularization parameter *α* = 0.5 in RLS, and set the number of nearest neighbors *K* = 5 in MLKNN^23^. In addition, we adopted the L2-norm when calculating the proximity measure in Equation 10.

We adopted the Area Under the Precision-Recall curves (AUPR) as the measuring metric for DDI prediction, because the number of drugs interacting with *d*_*j*_ (positive instances) is significantly less than that of drugs not interacting with *d*_*j*_ (negative instances) in each *d*_*j*_-specific classification. In such a case, AUPR performs a greater penalty on highly-scored false positive instances^40,41^ that the Area Under the Receiver Operating Characteristic Curve (AUC), which tends to generate an over-optimistic measure.

### Comparison between LCM and state-of-the-art approaches

**Figure 3:**
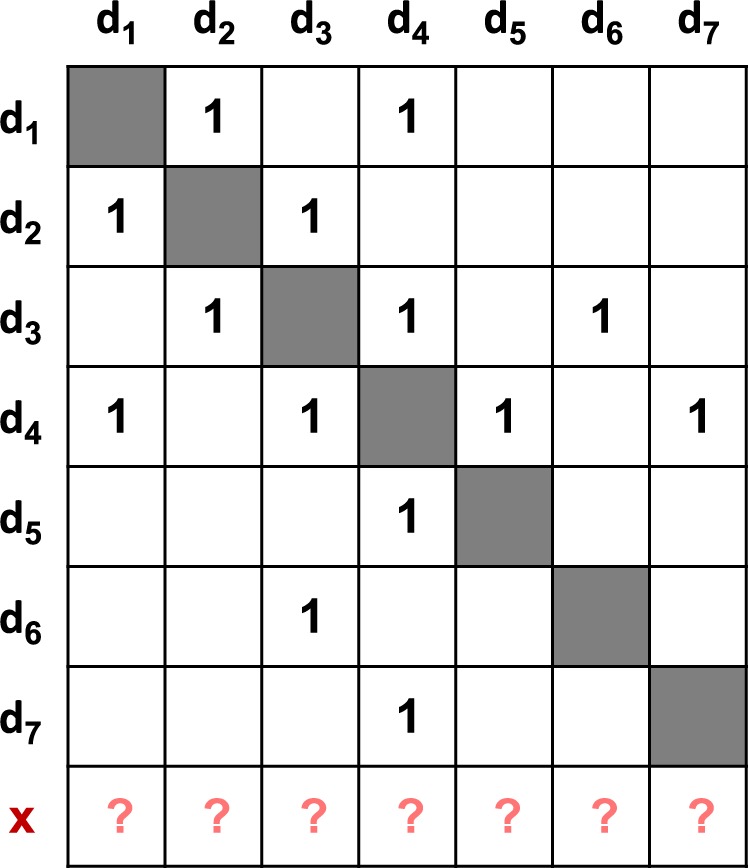
Illustration of hold-out cross validation. Eight drugs having known DDIs are randomly split into a training set and a testing set. The former contains seven training drugs (denoted as d1, d2, …, d7) while the latter contains only one testing drug x. The pairwise DDIs between the training drugs are organized into an interaction matrix, of which the cells marked with ‘1’s denote the interactions between the training drugs and the cells without mark denote non-interactions. The real interactions between ‘x’ and the training drugs are removed and marked with ‘?’ (See also Fig. 1). In the round of CV, the task is to deduce how possibly the testing x interacts with the training drugs one by one. The procedure is repeated until all the eight drugs are taken as the testing drugs in turn.

We first made a fair comparison with three state-of-the-art approaches, GCM^27^, NS^25^ and LP^28^. During the comparison, we performed the exactly same rounds of hold-out CV as those used by Zhang *et al*.^28^. In each round of hold-out CV, a fixed percentage (e.g. 25% hold-out ratio) of drugs were randomly selected as the testing drugs and all the DDIs associated with them are removed as well for validation. The remaining drugs were used as the training drugs and their pairwise DDIs were used to train predictive approaches. A toy diagram of hold-out CV is shown in Fig. 3. In addition, since GCM uses SVM as its classifier, we adopted SVM when implementing our LCM.

Such a round of CV with a specific hold-out ratio was repeated 50 times under 50 different random seeds^28^ and its result over the 50 repetitions was reported by the average of AUPR values, which were measured in all the rounds of the hold-out CV respectively. Totally, we performed five rounds of CV under 15%, 25%, 50%, 75% and 85% hold-out ratios (Table 1). The comparison reveals two observations: (1) all the local models, including NS, LP, and LCM, are better than GCM, because local models contain the topological information of DDI network whereas GCM does not; (2) LCM is remarkably superior to those state-of-the-art approaches with 6~22% improvement in terms of AUPR.

**Table 1:** Comparison under different ratios of hold-out cross validation (AUPR).

Approaches	Hold-out Ratio				
	*15%*	*25%*	*50%*	*75%*	*85%*
GCM (SVM)	0.5902	0.5871	0.5796	0.5776	0.5721
NS	0.7010	0.6992	0.6955	0.6986	0.6899
LP	0.7292	0.7282	0.7052	0.6736	0.6501
LCM (SVM)	**0.8078**	**0.8046**	**0.7824**	**0.7376**	**0.7117**

Secondly, to elucidate LCM’s advantage further, we compared our LCM with GCM, of which both apply SVM to perform DDI prediction, in terms of training time. We run LCM and GCM^27^ under those different hold-out ratios respectively in the computer, which is equipped with Intel 4700MQ (2.40 GHz) and 64-bit Windows 7 (Home Premium). Considering the fact that running of GCM cannot be performed with too many training instances (116,886 and 90,951 respectively) in the cases of 15% and 25% hold-out ratios, we randomly sampled the same number of training instances (40,470) as that in the case of 50% hold-out. Hence, the running time in such two scenarios of hold-out CV is approximately the same as that in the scenario of 50% hold-out CV. The results listed in Table 2 show that LCM runs significantly faster than GCM (with the same classifier, SVM), even subsampling is adopted.

**Table 2:** Comparison between LCM and GCM according to running time (seconds).

Approaches	Hold-out Ratio				
	*15%*	*25%*	*50%*	*75%*	*85%*
GCM (SVM)	50.195	48.989	49.078	23.843	4.044
LCM (SVM)	**7.954**	**6.356**	**2.047**	**0.349**	**0.168**

We further made a theoretical investigation about computational complexity. The computational complexity of SVM falls into the range of [*O*(*n*^2^), *O*(*n*^3^)], where *n* is the number of training instances. For *m* known drugs, GCM takes *m*(*m* + 1)/2 drug pairs as the training instances, while our approach only takes *m* drugs as the training instances in each of *m* classification tasks. Thus, the computational complexity of GCM is larger and in the range of the closed interval [*O*(*m*^4^), *O*(*m*^6^)]. In contrast, the computational complexity of LCM is bounded by [*O*(*m* × *m*^2^), *O*(*m* × *m*^3^)]. Therefore, in terms of computational complexity, LCM outperforms GCM.

Thirdly, to illuminate why LCM achieve the better prediction than NS and LP, we performed an additional investigation by leave-one-out cross-validation (LOOCV). We took one drug as the only one testing drug and the remaining drugs as the training drugs in each round of LOOCV. For the known drug *d*_*i*_ of interest, we first ranked the testing drug *d*_*x*_ by its predicted score, which indicates how likely *d*_*x*_ interacts with *d*_*i*_. For m known drugs (m = 568 here), *d*_*x*_ obtains m predicted scores. The higher the score is, the lower the value of rank is, and the higher the occurring chance of a DDI is. Usually, the top-n ranks of drug pairs are regarded as potential DDIs. We then calculated the correlation between the ranks and the degrees of all the known drugs. For drug *d*_*i*_, its degree is the number of other known drugs interacting with it. Finally, we repeated the procedure until each of the drugs were taken as the testing drug in turn and recorded the average value of the correlations obtained in all the rounds of LOOCV.

If such a correlation is significantly high, we say that the predictive model can be replaced by a degree only-based model. Thus, we investigated whether the ranks achieved by the predicting approaches are strongly correlated with the degrees of drug nodes in a DDI network. Considering that the relationship between the rank and the degree could be non-linear, we adopted Spearman’s correlation to assess it. Our investigation shows that the Spearman correlations of NS and LP are up to 0.998 and 0.983, whereas that of our LCM is 0.851. The extremely high correlations (>0.98) of both NS and LP indicate that they tend to recommend those drugs having many known DDIs as the interacting partners for a newly queried drug. The comparison reveals that the prediction achieved by a degree-related model would be greatly approximate to those achieved by NS and LP, but significantly different from that achieved by LCM.

The underlying reason is that both NS and LP involve the multiplication, which is correlated to the sum of the pairwise similarities between other existing drugs interacting with *d*_*i*_. As a result, their predictions are dependent on the number of positive instances (existing drugs interacting with *d*_*i*_) when predicting how possibly a newly-given drug *d*_*x*_ interacts with an existing drug *d*_*i*_. As a result, both of them have the degree-induced bias that leads their prediction to tend to rank the pairs between a newly-given drug and the drugs having many DDIs with top priorities. By contrast, the multiplication involved in LCM is usually related to the similarity matrix and a few of instances supporting the discriminate boundary in the case of SVM. Consequently, LCM only depends on the positive instances and the negative instances, of which both are located on the discriminate boundary, such that it is able to relax or minimize this bias.

Furthermore, we made a case study to show how the bias affects the prediction and demonstrate the ability of our LCM to relax the bias. We focused on the drug ‘Amoxapine’ which interacts with 7 known drugs having meanwhile different numbers of DDIs. We removed the interactions of ‘Amoxapine’ and predicted its interacting drugs. In an ideal prediction, it is anticipated that the ranks of the drug pairs between ‘Amoxapine’ and its interacting partners should be <= 7. We then extracted two of its interacting partner drugs, ‘Paroxetine’ and ‘Fluvoxamine’, which have the most and the least numbers of DDIs (444 and 101) respectively, and checked the real prediction achieved by NS, LP, and LCM. For the pair of ‘Paroxetine’ and ‘Amoxapine’, NS and LP generate rank 25 and rank 22 respectively whereas our LCM gives rank 4. Thus, our LCM generates the correct prediction (rank < 7) but they cannot. For the pair of ‘Fluvoxamine’ and ‘Amoxapine’, NS and LP give 366 and 361 whereas our LCM gives 204. Even all these approach cannot give a correct prediction, our LCM still gives a significantly higher rank than both NS and LP for the queried drug pairs. Similar predictions were able to be found in other cases. Consequently, our LCM is able to relax such a degree-induced bias.

### Validation of LCM-DS

In this section, we shall first show how the factors, including the posterior probabilities (${{\bf{y}}}_{n}^{x}$) directly output by a classifier, the proximity between them and the reference profiles $(||{{\bf{R}}}_{k}^{n}-{{\bf{y}}}_{n}^{x}||)$ of the training instances, and the classifier weight (*w*_*k*_(*n*)), affect the performance of LCM-DS respectively.

**Figure 4:**
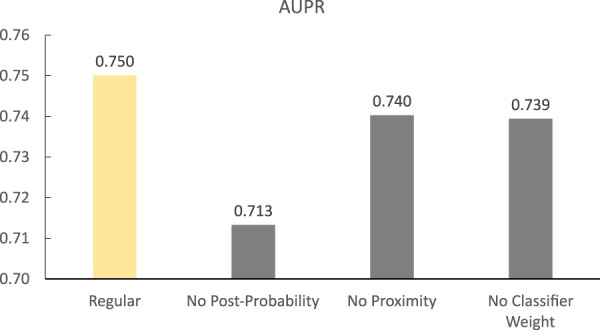
The influence of three factors in LCM-DS under the 85% ratio of Hold-Out Cross Validation with 50 Repetitions. The first label, ‘Regular’, denotes the regular LCM-DS without removing any factors. The other labels denote the variants of LCM-DS, which have no post-probability factor, no proximity factor and no classifier weight factor respectively.

To investigate the influence of these three factors, we built three variants of LCM-DS, of which each variant has the lack of a unique factor respectively. Then, we run and compared them with the regular LCM-DS (Fig. 4). The comparison shows that the lack of any of them decrease the predicting performance and the absence of the posterior probability factor causes the biggest decrement.

We made a case study to demonstrate the importance of the factors. Two drugs, ‘Prostacyclin’ and ‘Amikacin’, were chosen to investigate the predicted scores, which indicate how likely these two drugs interact with the training drugs. We sorted the predicted scores to rank the drug pairs, in which their partners are the training drugs, and reported the average ranks of the positively labeled drug pairs (Table 3). The less, the better.

**Table 3:** Comparison between LCM-DS and its variants in terms of average rank.

Drug	Average Rank			
	LCM-DS	No Post-probability	No proximity	No Classifier Weight
Prostacyclin	**90.16**	104.94	92.23	94.39
Amikacin	**52.79**	60.45	58.80	53.23

Three observations on these two drugs can be drawn: (1) all these factors contribute to the prediction because the absence of any of them increase the average ranks of DDIs for the selected drugs; (2) the factor of post-probability plays, as anticipant, the most important roles in LCM-DS because its absence causes the biggest increment of average ranks; and (3) LCM-DS integrating them achieves the best performance because it generates the smallest average ranks. Totally, the comparison demonstrates that LCM-DS is an effective fusion rule, which is able to integrate all the individual factors contributing to the prediction to obtain a better prediction.

**Figure 5:**
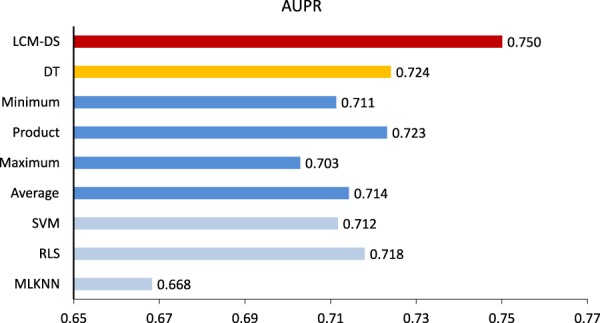
Comparison with Individual Classifiers and Classical Fusion Rules under the 85% ratio of Hold-Out Cross Validation with 50 Repetitions.

Moreover, we made a deeper investigation on LCM-DS by comparing it with both its member classifiers and classical fusion rules. The member classifiers are MLKNN, RLS, and SVM. The classical fusion rules include four unsupervised fusion rules (i.e. Average, Maximum, Product, Minimum) and one supervised fusion rule (Decision Template, DT^32^). Three individual classifiers were implemented under the framework of LCM and integrated into LCM-DS. In details, the similarity-based version of MLKNN was originally implemented by our previous work^23^, which developed an approach for predicting drug-target interactions. RLS was directly implemented by Octave codes. The implementation of SVM was by compiling and building the source codes of LibSVM^30^ into the Octave interface. All the fusion rules were also implemented by Octave codes. See also Section 2.3 for more technical details about the classifiers and see also Section 2.4 for more technical details about the fusion rules. We performed 85% hold-out CV again in the comparison (Fig. 5).

The comparison demonstrates that (1) the performance of individual classifiers varies and RLS achieves the best classifiers in this case of hold-out CV; (2) Former fusion rules may (e.g. Product, DT) or may not (e.g. Average, Maximum, Minimum) outperform individual classifiers; (3) DS wins the best among both member classifiers and classical fusion rules with the significant improvement. In summary, the proposed supervised DS-based fusion rule is effective.

## Discussion

DDIs frequently induce adverse drug reactions or occasionally facilitate better drug co-prescriptions. DDI identification before making clinical medications is critical but bears a high cost in clinics. Computational approaches have exhibited their ability on screening DDI candidates among a large number of drug pairs by utilizing preliminary characteristics of drugs. However, global model-based approaches are usually slow and neglect the topological structure of a DDI network, while local model-based models have the degree-induced bias.

To address these two issues, we have presented a novel local classification-based model (LCM) in the scenario of predicting DDI candidates for new drugs, which have no existing DDI with known drugs. For a specific drug having known DDIs, an LCM treats drugs having and having no interaction with it as positives and negative instances respectively, and trains a set of small-size classifiers to discriminate how likely a newly-given drug interacts with the drug of interest. Compared with the global classification-based model, LCM shows the advantages of theoretically faster running and practically better performance. Compared with two other local model-based approaches (naïve similarity-based and label propagation-based approaches), LCM is able to relax their intrinsic bias because the prediction for a new drug depends on the distributions or discriminant boundaries of positive instances and negative instances in the feature/similarity space.

More importantly, to address the issue that computational approaches lack an effective ensemble method to combine results from multiple predictors, we have designed a novel supervised fusion algorithm (LCM-DS) to aggregate the outputs of multiple classifiers for an unlabeled instance based on the Dempster-Shafer theory of evidence. Our LCM-DS integrates three factors from multiple classifiers, including the posterior probabilities output by individual classifiers, the proximity between the decision profiles of given instances and the reference profiles, as well as the quality of the reference profiles, which jointly contribute to the final decision.

Finally, both the experiments of DDI prediction and the case study demonstrate that the present LCM outperforms three state-of-the-art approaches, including one global model-based approach and two local model-based approaches, and its fusion version, LCM-DS, is superior to both all of its member classifiers and five classical fusion algorithms.

In the coming future, we shall improve our approaches in two aspects. First, LCM is of a supervised learning model, which treats unknown drug pairs as negative instances. In fact, a few of unknown drug pairs could be DDIs. Thus, a semi-supervised learning model^6^ or a one-class learning model should be considered. Secondly, other pre-existing knowledge should be considered in the proposed LCM-DS. Especially, the essence of DDI is strongly correlated with drug-binding proteins, such as drug targets and enzymes, which attend in different pathways. Thus, the integration of drug target-based^21,22,42,43^ and/or pathway-based^7^ similarities into the current similarities would be helpful to improve DDI prediction and even to reveal the underlying mechanism of DDI occurrence. In addition, because our LCM-DS actually provides an effective framework for combining decisions from different pre-existing sources, it can be easily applied in similar areas (e.g. lncRNA-disease association prediction^44^).
